# Anlotinib inhibits cervical cancer cell proliferation and invasion by suppressing cytokine secretion in activated cancer-associated fibroblasts

**DOI:** 10.3389/fonc.2024.1412660

**Published:** 2024-08-13

**Authors:** Yaozu Xiong, Xiaoting Xu, Xilei Zhou, Yusuo Tong, Changhua Yu

**Affiliations:** ^1^ Department of Radiation Oncology, Huai’an First People’s Hospital, Nanjing Medical University, Huai’an, China; ^2^ Department of Radiation Oncology, The First Affiliated Hospital of Soochow University, Suzhou, China

**Keywords:** CAFs, anlotinib, cervical cancer, IL-6, IL-8

## Abstract

**Objective:**

The aim of this study was to investigate whether anlotinib could exert an inhibitory effect on the proliferation and invasion of cervical cancer cells by inhibiting cytokines secreted by activated cancer-associated fibroblasts (CAFs).

**Methods:**

CAFs were isolated from cervical cancer tissues and experimentally studied *in vivo* and *in vitro*. Molecular biology experimental methods were used to verify whether anlotinib could inhibit the pro-carcinogenic effects of CAFs derived from cervical cancer tissues.

**Results:**

CAFs promote the proliferation and invasion of cervical cancer cells. Anlotinib inhibited the activation of CAFs and suppressed the promotion of cervical cancer cells by CAFs. Anlotinib inhibited the expression of multiple cytokines within CAFs and suppressed the release of interleukin (IL)-6 (IL-6) and IL-8. *In vivo* studies have shown that anlotinib diminished the growth of xenografted cervical cancer cells, and treatment in combination with docetaxel had an even more significant tumor growth inhibitory effect.

**Conclusion:**

Anlotinib inhibits the pro-cancer effects of CAFs by suppressing the activation of CAFs and the secretion of pro-cancer cytokines. Our findings suggest that the combination of anlotinib and docetaxel may be a potential strategy for the treatment of refractory cervical cancer.

## 1 Introduction

Globally, cervical cancer is the fourth most common female malignancy, with more than 600,000 new cases and more than 341,000 deaths worldwide in 2020. Cervical cancer remains a major public health problem affecting women, especially in developing countries with fewer resources (1). In recent years, thanks to the progress of surgery and radiotherapy technology, the therapeutic effect of cervical cancer has been partially improved, but the overall prognosis of women with metastatic or recurrent diseases is still very poor (2). Therefore, it is necessary to find new treatment strategies to improve the prognosis of cervical cancer.

The study concluded that cancer progression is the result of ongoing development and crosstalk between different cell types within the tumor micro-environment (TME) (3). Stromal cells in TME mainly include fibroblasts, macrophages, inflammatory cells, endothelial cells and epithelial cells (4). Cancer-associated fibroblasts (CAFs) has been reported to be biologically and clinically important in TME, where it is involved in stimulating the proliferation and progression of cancer cells and promotes malignant behaviors such as proliferation, invasion, metastasis, and resistance to treatment through interacting signaling pathways (5–7). Activated CAFs in most tumors can be involved in the regulation of TME through autocrine or paracrine secretion of a large number of cytokines, growth factors and chemokines at different stages of tumor progression. The secretion products of CAFs mainly include various regulatory factors such as interleukin(IL)-1 (IL-1), IL-6, IL-8, IL-10, transforming growth factor-β (TGF-β), tumor necrosis factor-α (TNF-α), C–X–C motif chemokine ligand 1 (CXCL1), CXCL12 and stromal cell-derived factor 1 (SDF1). These cytokines act on tumor cells and participate in tumorigenesis and development, and can increase the invasiveness of co-cultured cancer cells (8–10). These findings suggest that targeting CAFs may be a novel strategy to control the proliferation and invasiveness of cervical cancer cells caused by activated CAFs.

Anlotinib is a novel oral multi-target tyrosine kinase inhibitor (TKI), which can effectively inhibit vascular endothelial growth factor receptor (VEGFR), platelet-derived growth factor receptor (PDGFR), fibroblast growth factor receptor (FGFR) (11). Anlotinib was found to alleviate liver fibrosis in rats by down-regulating the expression of α-smooth muscle actin (α-SMA) and type I collagen in hepatocytes (12). It can also significantly improve lung function in a mouse model of lung adenocarcinoma combined with pulmonary fibrosis, reduce collagen content in lung tissue, and inhibit the growth of lung tumors in mice (13). The aim of this study was to verify whether anlotinib inhibits CAFs derived from cervical cancer tissues. To our knowledge, no studies have been conducted on the effects of anlotinib on CAFs in cervical cancer tissues. Therefore, we investigated the effects of anlotinib on cervical cancer-derived CAFs that promote malignant behaviors of cervical cancer cells, such as proliferation and invasion. Furthermore, by using an *in vivo* xenograft animal model with co-implanted CAFs and cervical cancer cells, we evaluated the therapeutic efficacy of combining conventional chemotherapeutic agents (e.g., docetaxel) with anlotinib to target inhibition of activated CAFs.

## 2 Methods

### 2.1 Cell cultures and cell isolation

**Figure 1:**
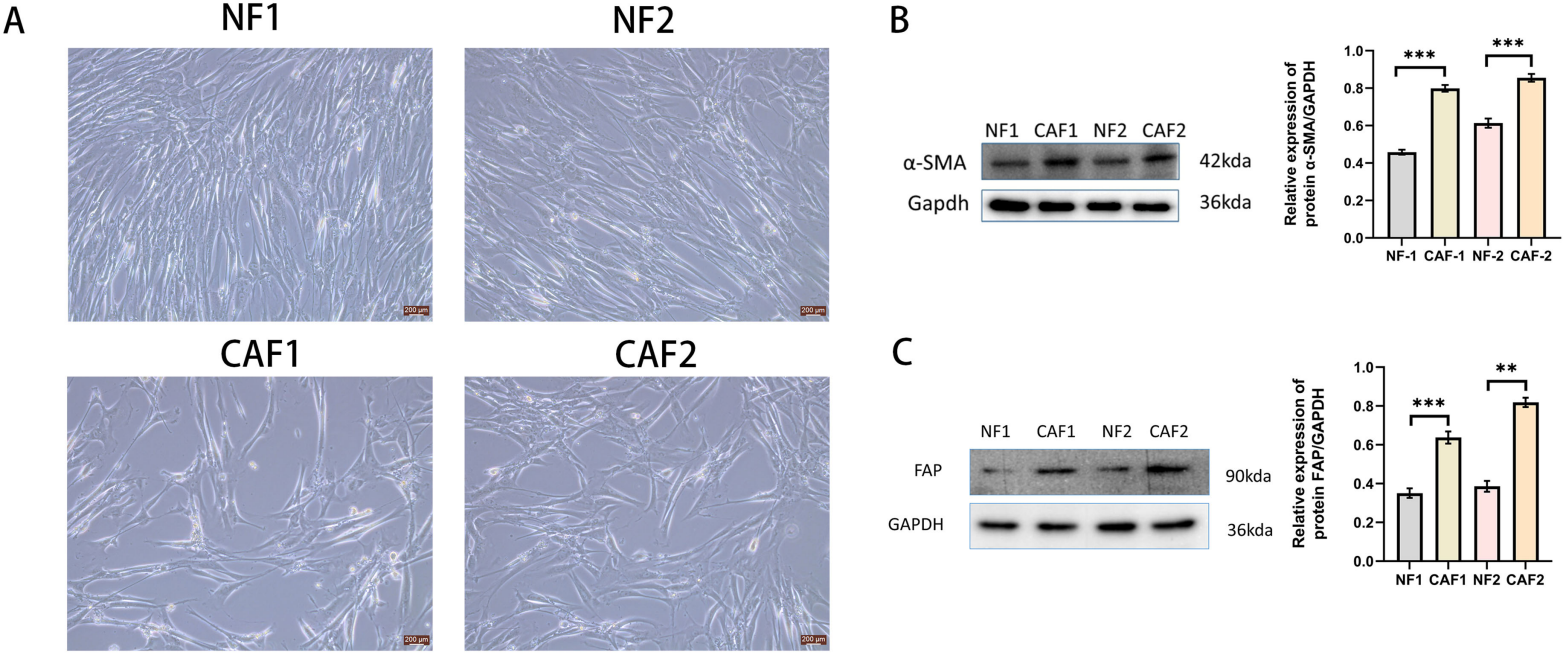
Successfully isolated CAFs from cervical cancer tissue. **(A)** Representative pictures of NFs and CAFs in paraneoplastic and cervical cancer tissues(Scale bar: 200 um). **(B)** Western blotting examination of α-SMA protein expression in NFs and CAFs(n=3). **(C)** Western blotting examination of FAP protein expression in NFs and CAFs(n=3). **p<0.01, ***P<0.001.

We established primary CAFs (CAF1 and CAF 2) and NFs (NF1 and NF2) from surgically resected cervical cancer tissues and paracancerous tissues from Huai’an First Hospital of Nanjing Medical University by absorption isolation method (14). Briefly, cervical cancer tissues were minced into 1-5 mm^3^ pieces and incubated in Dulbecco’s modified Eagle’s medium (DMEM)(Gibco, Carlsbad, CA, USA), supplemented with 20% fetal bovine serum(FBS)(Gibco, Carlsbad, CA, USA), 1% penicillin-streptomycin(Gibco, Carlsbad, CA, USA), and 1 ng/mL basic fibroblast growth factor(bFGF),(#42430,Cell Signaling Technology) and maintained in a constant temperature incubator at 37°C, 5% CO_2_ until the cells were attached to the culture dish. Unattached cells were removed and the supplemental medium was repeated twice weekly. We confirmed that CAFs exhibited fibroblast-like morphology and positive α-SMA and FAP expression by Western blotting (**Figure 1**). We used CAFs with passages between 4 and 8 for subsequent experiments. Establishment of CAFs from surgically excised cervical cancer tissues was approved by the Ethics Institutional Review Board of Huai’an First Hospital, Nanjing Medical University, China (Approval number:20220189). The human cervical cancer cell lines Hela and Siha used in this study were purchased from the Shanghai Cell Bank of the Chinese Academy of Sciences. Hela and Siha cells were cultured in DMEM supplemented with 10% FBS and 1% penicillin–streptomycin (Gibco, Carlsbad, CA, USA), and maintained at 37°C in a humidified incubator with 5% CO_2_ atmosphere.

### 2.2 Anlotinib

Anlotinib was purchased from Chengtai Tianqing Pharmaceutical Co. (Lianyungang, Jiangsu, China). Anlotinib was dissolved in dimethyl sulfoxide (DMSO) (Sigma-Aldrich, St. Louis, MO, USA) and stored at -20°C, protected from light, and configured to final concentrations of 0,4,8,16,32 umol/L for *in vitro* experiments. Anlotinib was dissolved in Tween-80 (Sigma-Aldrich, St. Louis, MO, USA) solution at a final concentration of 1.5 mg/kg for *in vivo* experiments.

### 2.3 Preparation of conditioned media

CAFs were cultured in DMEM supplemented with 20% FBS and 1 ng/mL bFGF until they reached 90% confluence. Next, the medium was replaced with serum-free DMEM with 5 ng/mL recombinant transforming growth factor β(TGF-β) (R&D systems, Minneapolis, USA) and the cells were cultured for an extra 48 h. At the completion of the incubation, the CAF-CM was collected and centrifuged at 1500 g for 10 min. NFs were cultured in DMEM supplemented with 20% FBS and 1 ng/mL bFGF until they reached 90% confluence. Then, the medium was replaced with serum-free DMEM and centrifuged after 48 h of incubation to obtain NF-CM. To evaluate the effects of anlotinib, CAFs were cultured in serum-free DMEM containing 4 uM anlotinib for 48 h and anlotinib-treated CAF-CM was collected.

### 2.4 Cell proliferation assay

Cell proliferation was determined using the Cell Counting Kit-8 (Dojindo, Kyushu Island, Japan). CAFs and cervical cancer cell lines were inoculated and cultured in 96-well plates, replacement of the medium with serum-free medium containing anlotinib at a concentration of 0 - 32 uM. To assess cell proliferation after stimulation with CM, the culture medium of cervical cancer cells was replaced with CAF-CM or anlotinib-treated CAF-CM after overnight incubation. Cell proliferation levels were assayed after 48 h. The absorbance of each well was measured at 450 nm using a spectrophotometer (Bio-Rad, Hercules, CA, USA).

### 2.5 Clone formation assay

Clone formation assays was used to assess the effect of CAFs on the proliferation of cervical cancer cells. Cervical cancer cells in logarithmic phase were digested with trypsin and inoculated into 100 mm culture dishes. After cell adhesion, cervical cancer cell medium was replaced with CAF-CM and incubated continuously for 14 days to form cell colonies.

### 2.6 EDU labeling assay

Detection of the effect of CAFs on the proliferation of cervical cancer cells using Cell-Light EDU Apollo 488 *in vitro* imaging kit (Ribobio, China). Cervical cancer cells were cultured in DMEM or CAF-CM for 48 h. Then, tumor cells were incubated with EDU-labeled medium. After 2 h of incubation, the cells were fixed with 4% paraformaldehyde and permeabilized with 0.5% Triton X100. Then, the cells were stained with Apollo 488 for 30 min. Subsequently, the cells were restrained with Hoechst 33342 and images were captured for analysis using confocal microscopy (Nikon Eclipse Ti, Japan).

### 2.7 Invasion assay

Cell invasion assay using 24-well Corning^®^ BioCoat™ Matrigel Invasion Chambers (Corning, NY, USA). Cervical cancer cells were inoculated in the upper chamber in FBS-free medium and the lower chamber was filled with medium supplemented with 3% FBS and containing 4 uM of anlotinib, CAF-CM, or anlotinib-treated CAF-CM.

### 2.8 Protein extraction and western blotting

CAFs were treated with concentrations of 0, 4,8,16 uM of anlotinib for 72 h, followed by protein extraction. Cervical cancer cell lines were treated with anlotinib, CAF-CM, and anlotinib-treated CAF-CM for 12 h, and then proteins were extracted. Cervical cancer cell lines were treated with 10 ng/ml recombinant IL-6 protein (R&D systems, Minneapolis, USA) and 10 ng/ml recombinant IL-8 protein (R&D systems, Minneapolis, USA) for 2 h, followed by protein extraction. According to the molecular weight of the target protein, the appropriate concentration of SDS-PAGE gel was selected for electrophoretic separation of the protein specimen, and the separated proteins were transferred to the polyvinylidene fluoride (PVDF) membranes (Merck Millipore), and then the membrane was closed using 5% concentration of skimmed milk for 1.5 h at room temperature, the primary antibody was incubated for 16 h at 4°C, and the secondary antibody was incubated for 1 h at room temperature, and the bands were visualized using the ECL kit (Beyotime, Jiangsu, China), and the chemiluminescence gel imager (Carestream Health, Inc., Rochester, NY, USA) to show the bands and a chemiluminescent gel imager (Carestream Health, Rochester, NY, USA) to observe the bands. The primary antibodies used in this study were anti-α-SMA mouse mAb (A2547, 1:1000, Sigma-Aldrich, St. Louis, MO, USA), phosphorylated Stat 3(p-Stat3) rabbit mAb (#9145, 1:1000, Cell Signaling Technology, MA, USA), Stat 3 mouse mAb (#9139S, 1:1000, Cell Signaling Technology), phosphorylated janus kinase-2 rabbit mAb (p-Jak2) (#3771,1: 1000, Cell Signaling Technology, Massachusetts, USA), Jak2 rabbit mAb(#3230, 1: 1000, Cell Signaling Technology), Fap rabbit mAb (#52818S, 1:1000, Cell Signaling Technology), Akt rabbit mAb(#4691, 1:1000, Cell Signaling Technology), Erk rabbit mAb(#4695, 1:1000, Cell Signaling Technology), Caspase3 rabbit mAb(#9662, 1:1000, Cell Signaling Technology), Cleaved Caspase3 rabbit mAb(#9664, 1:1000, Cell Signaling Technology) and anti-Gapdh mouse mAb(#2118, 1:1,000,Cell Signaling Technology) were used as loading controls.

### 2.9 Ultracentrifuge

The CAF-CM was ultracentrifuged at 110,000 g at 4°C for 70 min. The supernatant was collected after the first centrifugation. The precipitate was washed with phosphate-buffered saline, subjected to ultracentrifugation and resuspended in serum-free DMEM.

### 2.10 RNA extraction and qRT-PCR

Total RNA was extracted from cultured cells using TRIzol reagent (Invitrogen). qRT-PCR was performed using SYBR Premix Ex Taq II (Takara, Japan) according to the ABI 7500 Real-Time PCR System (Applied Biosystems, Foster City, CA, USA) manufacturer’s instructions. Each sample was performed in triplicate. GAPDH was used to normalize mRNA expression levels. All primers used in this study are listed in **Table 1**.

**Table 1:** Primer pairs used for qRT-PCR.

Genes	Primer pairs	
IL-1β	Forward	5’-ATGATGGCTTATTACAGTGGCAA-3’
	Reverse	5’-GTCGGAGATTCGTAGCTGGA-3’
IL-6	Forward	5’-ACTCACCTCTTCAGAACGAATTG-3’
	Reverse	5’-CCATCTTTGGAAGGTTCAGGTTG-3’
IL-8	Forward	5’-TTTTGCCAAGGAGTGCTAAAGA-3’
	Reverse	5’-AACCCTCTGCACCCAGTTTTC-3’
IL-10	Forward	5’-GACTTTAAGGGTTACCTGGGTTG-3’
	Reverse	5’-TCACATGCGCCTTGATGTCTG-3’
VEGFA	Forward	5’-AGGGCAGAATCATCACGAAGT-3’
	Reverse	5’-AGGGTCTCGATTGGATGGCA-3’
TNF-α	Forward	5’-CCTCTCTCTAATCAGCCCTCTG-3’
	Reverse	5’-GAGGACCTGGGAGTAGATGAG-3’
VCAM1	Forward	5’-GGGAAGATGGTCGTGATCCTT-3’
	Reverse	5’-TCTGGGGTGGTCTCGATTTTA-3’
IL-11	Forward	5’-CGAGCGGACCTACTGTCCTA-3’
	Reverse	5’-GCCCAGTCAAGTGTCAGGTG-3’
TGF-β	Forward	5’-CAATTCCTGGCGATACCTCAG-3’
	Reverse	5’-GCACAACTCCGGTGACATCAA-3’
GAPDH	Forward	5’-CCCTCTGGAAAGCTGTGG-3’
	Reverse	5’-AGTGGATGCAGGGATGATG-3’

### 2.11 Enzyme-linked immunosorbent assay

The levels of IL-6, IL-8, and VEGFA were detected using human Elisa IL-6 kit, human ELISA IL-8 kit, and human Elisa VEGFA kit (Quanticyto, neobioscience, Shenzhen, China). CAF-CM and anlotinib-treated CAF-CM were ultracentrifuged at 110,000 g for 70 min at 4°C, and the supernatant was collected. 10 μl of sample supernatant was added to the assay wells by mixing with 90 ul of diluent. Next, the kit instructions were followed and the final cytokines content was detected by measuring the absorbance at 450 nm.

### 2.12 *In vivo* experiment

**Figure 2:**
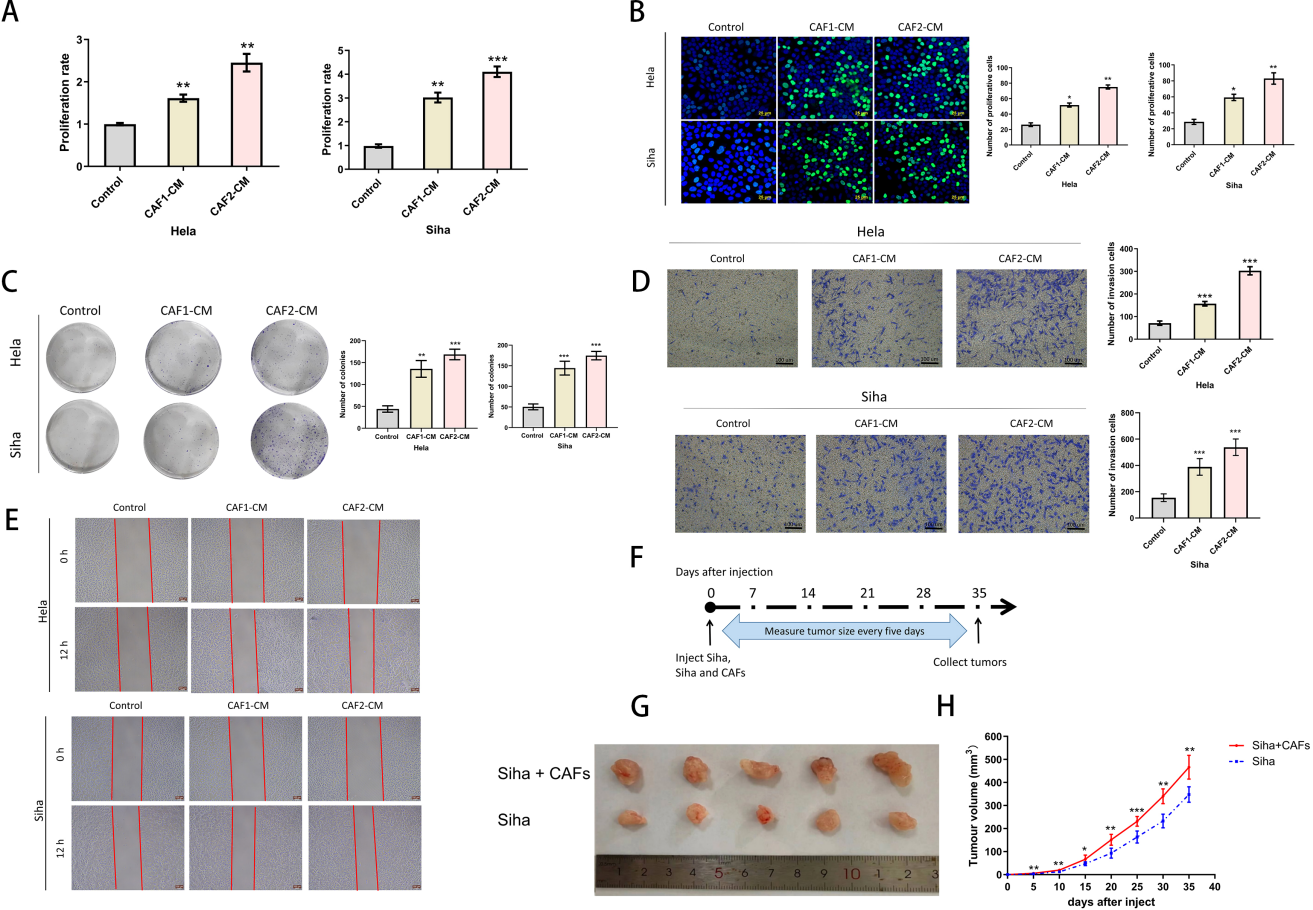
Effects of CAFs on the proliferation and invasive ability of cervical cancer cells. **(A)** CCK-8 assays verified that CAF-CM enhanced the proliferation of Hela and Siha (n= 5). **(B)** EDU assays verified that CAF-CM promoted the proliferation of Hela and Siha (n=5) (Scale bar: 25um). **(C)** Clone formation assays verified that CAF-CM promoted the proliferation of Hela and Siha (n=5). **(D)** The invasion assay verified that CAF-CM enhanced the invasive ability of Hela and Siha (n=5) (Scale bar: 100um). **(E)** Wound healing assays verified that CAF-CM enhanced the migration of Hela and Siha(n=5)(Scale bar: 200um). **(F)** Measurement and collection timeline of xeno-implanted tumors. **(G, H)** Tumor pictures and growth volumes of Siha and Siha plus CAF cells (n=5). *p<0.05, **P<0.01, ***P<0.001. Compared with the control group.

**Figure 3:**
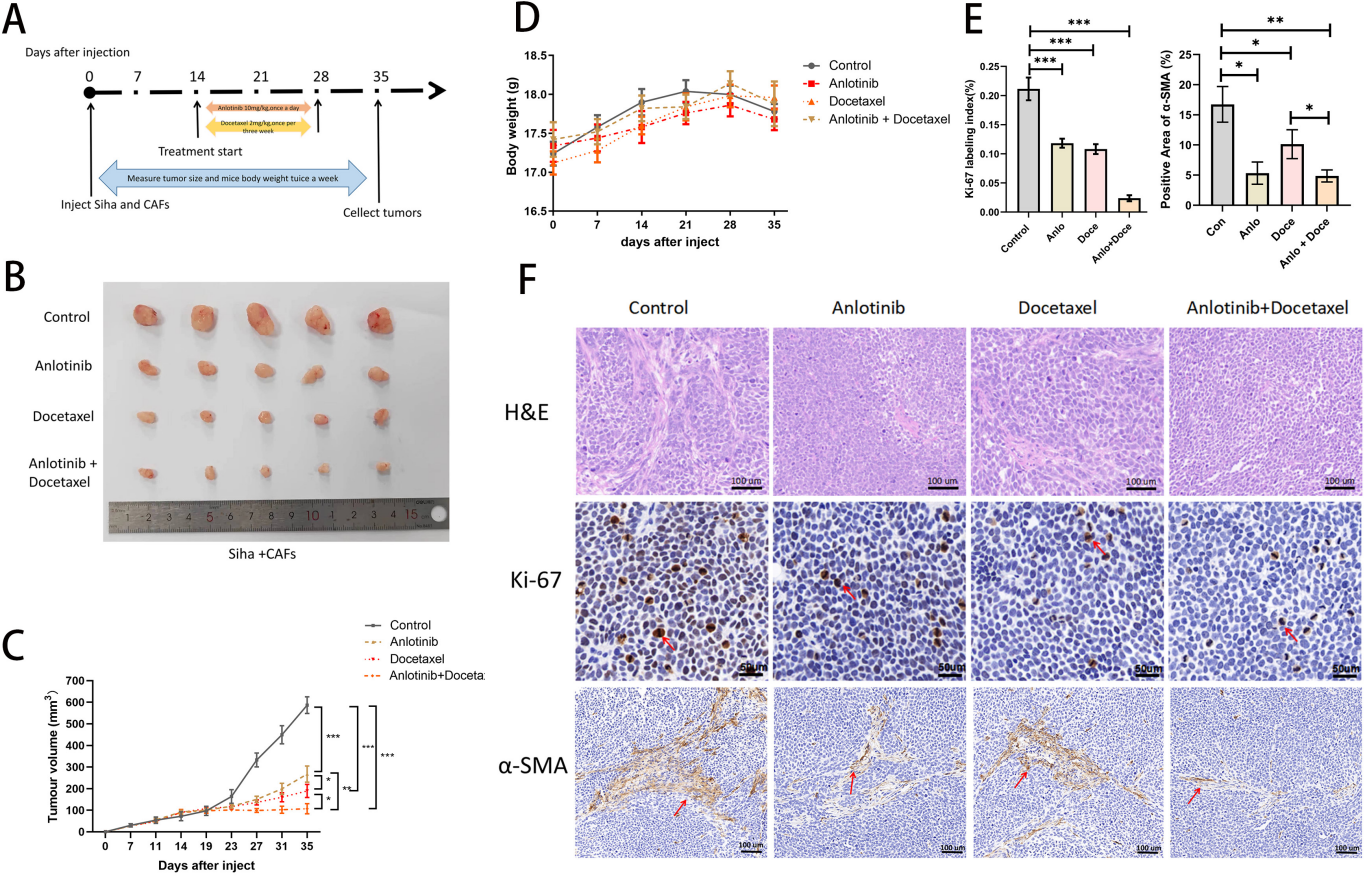
Anlotinib reduces tumor volume consisting of Siha plus CAFs and significantly inhibits tumor proliferation in combination with docetaxel. **(A)** Anlotinib and docextaxel treatment schedule. **(B)** Tumor growth curves showed that anlotinib plus docetaxel combination therapy maximally inhibited the growth of tumors composed of Siha + CAFs (n=5). **(C)** Photographs of tumors in the four treatment groups (control, anlotinib, docetaxel, and anlotinib + docetaxel). **(D)** Relationship between mouse body weight and treatment group in the xenograft experiments. There was no correlation between the body weight and treatment group (n=5). **(E)** Treatment with both anlotinib and docetaxel significantly reduced the number of KI-67-positive cells and α-SMA-positive expression areas in the tumor. Combined treatment with the two was even more effective (Anlo: Anlotinib group, Doce: Docetaxel group, Anlo+Doce: Anlotinib+Docetaxel group) (n=5). **(F)** Representative micrographs of H&E staining and Ki-67,α-SMA immunohistochemistry results for the four treatment groups (Scale bar: 50um and 100um). *P<0.05, **P<0.01, ***P<0.001. Compared with the control group.

To investigate the role of CAFs in an *in vivo* animal xenograft model, we compared the proliferation rates of Siha cells alone and Siha plus CAF cells. Siha cell suspension (5 × 10^6 cells) and Siha (5 × 10^6 cells) + CAF (1 × 10^6 cells) cell suspensions were injected bilaterally subcutaneously into the coeliac region of 7-weeks-old female nude mice, with each group containing 5 xenografts in each group. The experiments were performed according to the schedule given in **Figure 2F**. In addition, to analyze the effects of anlotinib in animals, we used a nude mouse xenograft model of Siha + CAFs cells. At 2 weeks after implantation, we randomized the mice into a control group, an anlotinib group, a docetaxel group, and an anlotinib plus docetaxel group. We administered 1.5 mg/kg of anlotinib orally 7 times per week and 50 mg/kg of docetaxel intraperitoneally 2 times per week to mice under isoflurane anesthesia. Each group contained 5 xenografts. The experiments were performed according to the schedule in **Figure 3A**. Tumor diameters and body weights were measured twice a week, and tumor volumes were calculated using the following formula: (S × S × L)/2, where S is the short diameter of the tumor and L is the long diameter of the tumor. To collect xenograft tumors, mice were anesthetized with isoflurane and euthanized by cervical dislocation. Tumors were evaluated microscopically using hematoxylin and eosin staining and immunohistochemistry. Mice had free access to water and food and were housed in cages and bedding free of specific pathogens under a 12 h light/dark regimen and controlled room temperature. All mouse experiments were performed according to the guidelines of the Laboratory Animal Center of the First People’s Hospital of Huai’an.

### 2.13 Hematoxylin and eosin staining

Tumor tissues were fixed in 4% paraformaldehyde for 24 hours and then paraffin embedded. Tissues 5um thick were cut to make pathological sections. After baking at 90° for 10 mins, the tissues were transparent in xylene, and alcohol was used for gradient hydration, then stained with hematoxylin and eosin, and sealed with neutral gum. The sections were observed under a light microscope and photographed.

### 2.14 Immunohistochemistry

The number of Ki-67-positive cells and the area rate of α-SMA-positive in tumor tissues was detected using immunohistochemistry. Cell number determination and rate of positively stained areas was performed using ImageJ 1.5 image analysis software (National Institute of Health, MD, USA). Anti-Ki-67 rabbit mAb (M7240, 1:200, Dako, Agilent Technologies, CA, USA).

### 2.15 Immunofluorescent

5 um xenograft tumor tissue was removed from paraffin and hydrated with gradient ethanol. After antigen repair was performed, the tissues were blocked with 5% bovine serum albumin (BSA) for 30 min at 37°C, and then incubated with the following primary antibodies overnight at 4°C. After washing with PBS, the tissues were incubated with Alexa-Fluor 488 and 555 donkey anti-rabbit/mouse secondary antibodies (1:500,beyotime, Jiangsu, China) for 2 h at room temperature. Finally, the cell nuclei were stained and sealed with anti-fluorescence quenching sealer (containing DAPI) (beyotime, Jiangsu, China). Images were taken by an Olympus confocal microscope (Olympus, Tokyo, Japan). The primary antibodies used in this study were α-SMA (1:500, ab5694, Abcam), IL-6 (1:200, ab233706, Abcam), IL-8 (1:200, ab289967, Abcam), VEGFA (1:500, ab52917, Abcam).

### 2.16 Statistical analysis

Data were statistically analyzed using the statistical software SPSS 23.0. GraphPad Prism 9.3 was used to create figures. Data for continuous variables were expressed as mean ± standard deviation (SD) representation. Differences between the two groups were estimated using t-test. Differences between the four groups were assessed using ANOVA and Tukey’s multiple comparison test. Differences between the two groups were analyzed using t-tests. Differences between the four groups were assessed using analysis of variance and Tukey’s multiple comparison test. All P < 0.05 or 0.01 or 0.001 were considered statistically significant.

## 3 Results

### 3.1 We successfully isolated CAFs from cervical cancer tissues and normal fibroblasts from paracancerous tissues

NFs were regular in size and morphology, showing a flat long spindle shape. CAFs were heterogeneous in size and morphology, and grew haphazardly (**Figure 1A**). The protein expression of α-SMA and FAP was low in NFs and high in CAFs (**Figures 1B, C**).

### 3.2 Cervical cancer-derived CAFs can promote the proliferation and invasion of cervical cancer cells

To confirm the effects of CAFs on cancer progression, we treated cervical cancer cell lines with CAF-CM, and the results showed that CAF-CM could promote proliferation (**Figures 2A–C**), invasion (**Figure 2D**), and migration (**Figure 2E**) of cervical cancer cell lines. The growth size of Siha cells co-implanted with CAFs in nude mice was significantly larger than that of Siha cells implanted in nude mice alone during the same growth cycle (**Figures 2G, H**).

### 3.3 Anlotinib inhibits the activation of CAFs

**Figure 4:**
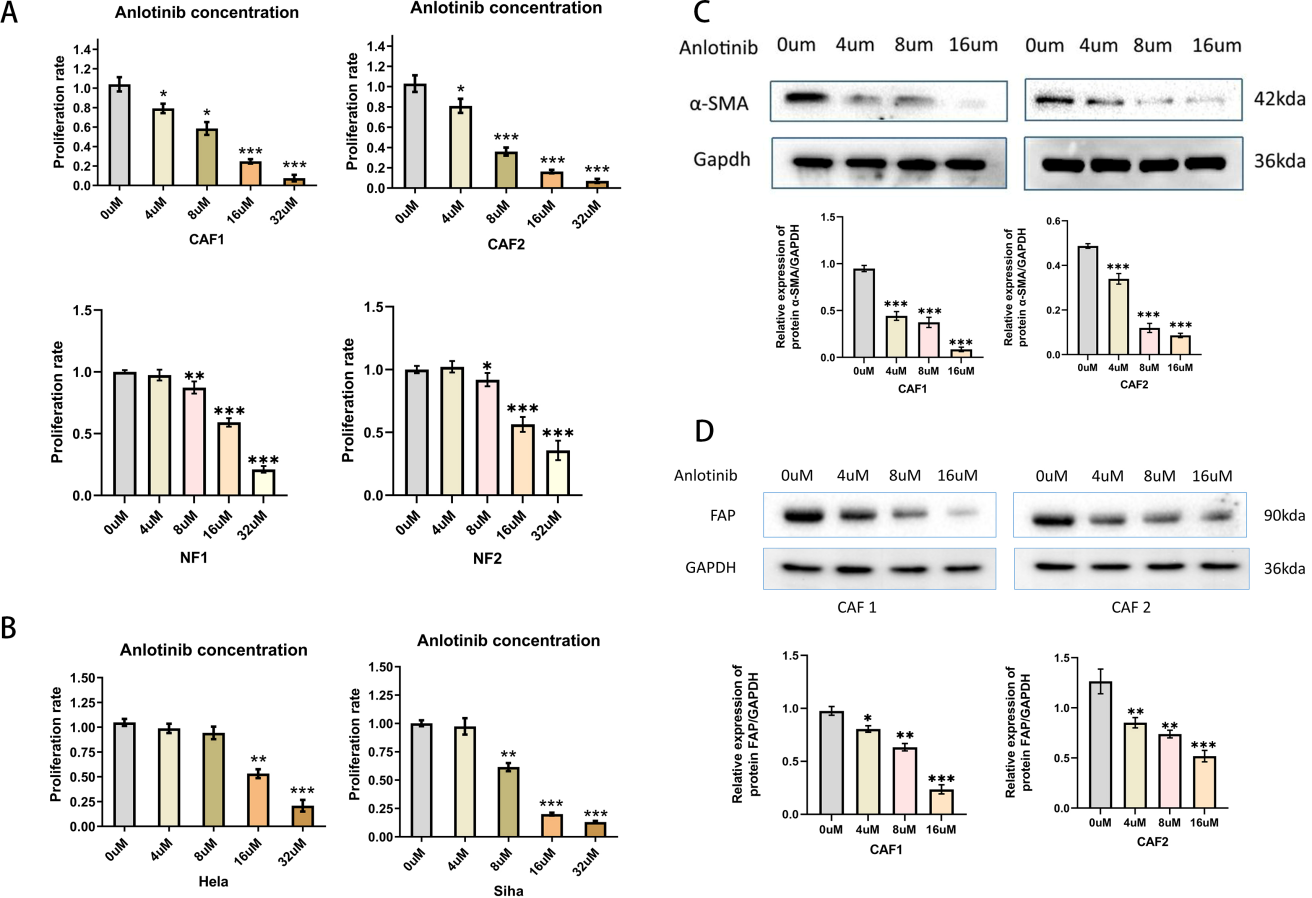
Effects of anlotinib on CAFs and Cervical Cancer Cells. **(A)** Anlotinib concentration-dependently inhibited the proliferation of CAFs. High concentrations of anlotinib inhibit the proliferation of NFs (n = 5). **(B)** Anlotinib inhibited proliferation of Hela and Siha only at high concentrations (n = 5). **(C)** The protein expression of α-SMA was evaluated by western blotting in anlotinib-treated CAFs(n=3). **(D)** The protein expression of FAP was evaluated by western blotting in anlotinib-treated CAFs (n=3). *P<0.05, **P<0.01, ***P<0.001. Compared with the 0uM group.

To verify the effect of anlotinib on CAFs, NFs and cervical cancer cell lines, we evaluated the effect of anlotinib on the proliferation of CAFs, NFs and cervical cancer cell lines. Anlotinib significantly inhibited CAFs proliferation in a concentration-dependent manner compared to control group (**Figure 4A**). On the other hand, the inhibitory effect of anlotinib on cervical cancer cell lines could be observed only at high concentrations above 8 uM (**Figure 4B**). Low concentrations (4 uM) of anlotinib did not inhibit the proliferation of NFs. When the concentration exceeded 8uM, the proliferation of NFs was inhibited by anlotinib (**Figure 4A**). In addition, anlotinib inhibited the protein expression of α-SMA and FAP, markers of activated CAFs, in CAFs cells in a concentration-dependent manner (**Figures 4C, D**).

### 3.4 Anlotinib could eliminate the proliferation and invasive ability of cervical cancer cell lines induced by CAFs

**Figure 5:**
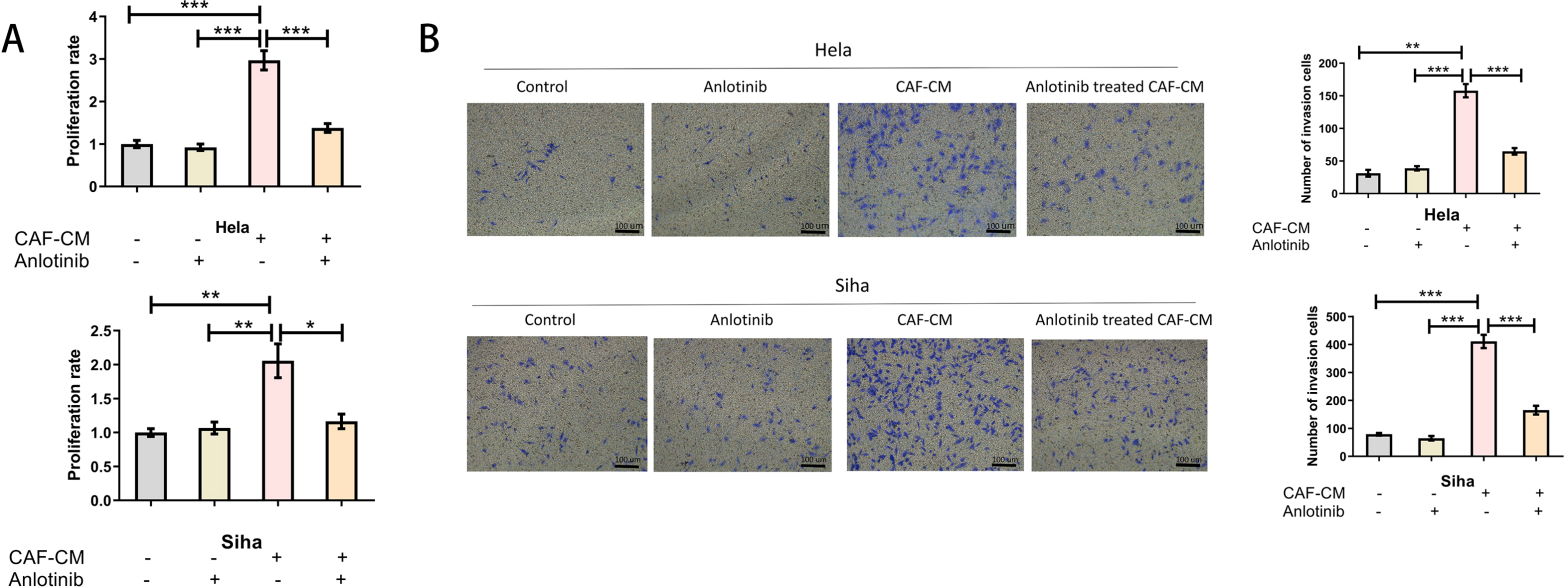
Anlotinib inhibits the promotion of cervical cancer cells by CAFs. **(A)** Treatment with anlotinib eliminated the proliferation-promoting effects of CAF-CM on Hela and Siha (n = 5). **(B)** Treatment with anlotinib decreased the invasion-promoting effects of CAF-CM on Hela and Siha (n = 5) (Scale bar: 100um). *P<0.05, **P<0.01, ***P<0.001. Compared with the control group.

To determine the effect of anlotinib on the malignant behavior of cervical cancer cell lines induced by CAFs, we investigated the proliferative and invasive capacity of cervical cancer cell lines using anlotinib-treated CAF-CM. Low concentrations (4 uM) of anlotinib had no significant direct effect on proliferation and invasion of cervical cancer cell lines, and CAF-CM increased proliferation and invasion of cervical cancer cell lines. In addition, treatment with a low concentration (4 uM) of anlotinib eliminated the proliferation-enhancing effects of CAF-CM (**Figure 5A**), while anlotinib was also observed to inhibit the invasion-enhancing effects of CAF-CM on cervical cancer cell lines (**Figure 5B**).

### 3.5 Anlotinib inhibits the expression and release of cytokines within CAFs

**Figure 6:**
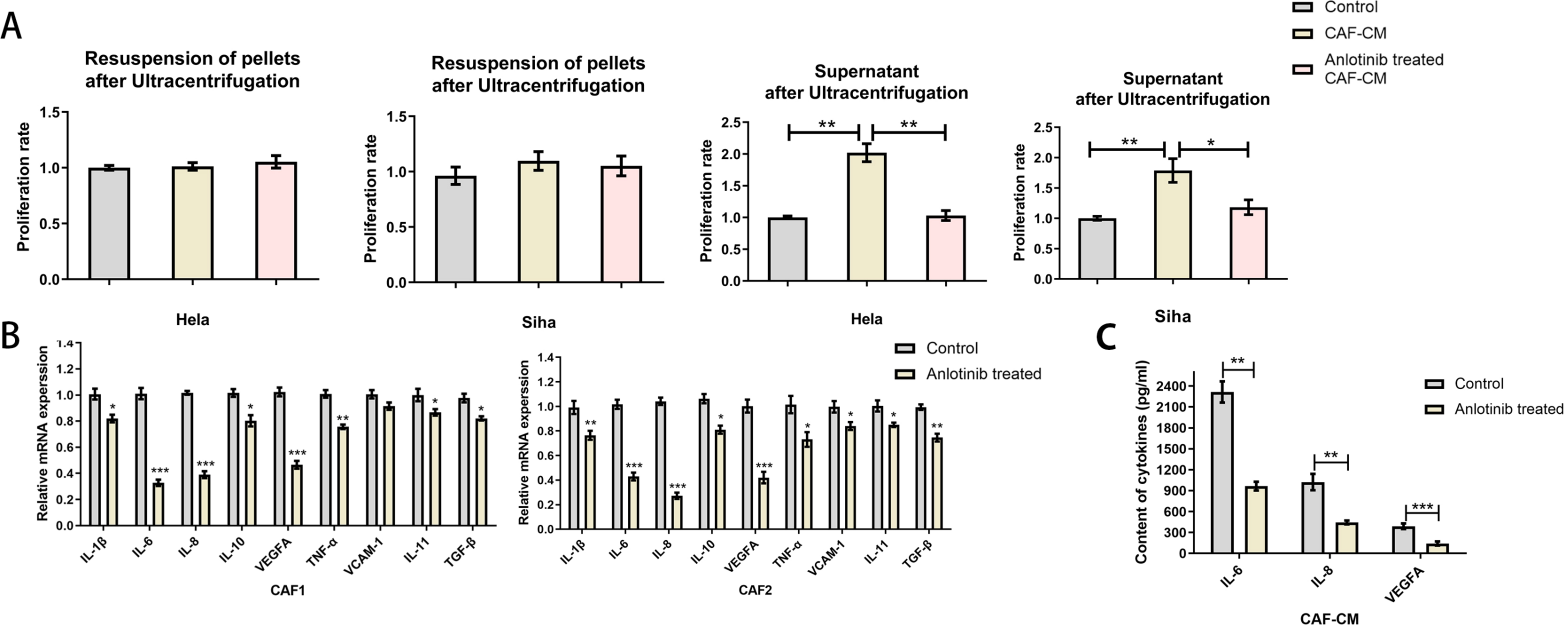
Anlotinib inhibits cytokines expression and secretion within CAFs. **(A)** Proliferation-promoting effects on Hela and Siha cells were observed only in the supernatant of CAF-CM after ultracentrifugation, which was eliminated by treatment with anlotinib (n=5). **(B)** Anlotinib inhibits some cytokines expression in CAFs (n=5). **(C)** Anlotinib inhibited IL-6,IL-8,VEGFA secretion within CAFs (n=5). *P<0.05, **P<0.01, ***P<0.001. Compared with the control group.

To clarify the factors associated with the inhibition of CAFs by anlotinib, we evaluated the soluble cytokines in the supernatant of CAF-CM and the precipitates in the lower fluid by ultracentrifugation. The supernatant of CAF-CM after ultracentrifugation had the same effect as the original solution in promoting the proliferation of cervical cancer cell lines, and the treatment with anlotinib eliminated the effect. On the contrary, ultracentrifugation followed by resuspension of the precipitates did not show significant pro-proliferative effects on cervical cancer cell lines (**Figure 6A**). To determine the effect of anlotinib on CAFs, we subsequently performed qRT-PCR analyses to evaluate differences in common cytokine gene expression between CAFs and anlotinib-treated CAFs. Treatment with anlotinib was found to inhibit the expression of multiple cytokine genes in CAFs, including IL-1β, IL-6, IL-8, IL-10,TNF-α, VEGF,VCAM-1 and IL-11, TGF-β, with a more pronounced inhibitory effect on IL-6, IL-8, and VEGF in particular (**Figure 6B**). It was further verified by an ELISA assay that treatment with anlotinib significantly inhibited the secretion of IL-6, IL-8, and VEGF in CAFs (**Figure 6C**).

### 3.6 Treatment with anlotinib inhibits the promotional effects of IL-6 and IL-8 on the proliferation and invasion of cervical cancer cell lines

**Figure 7:**
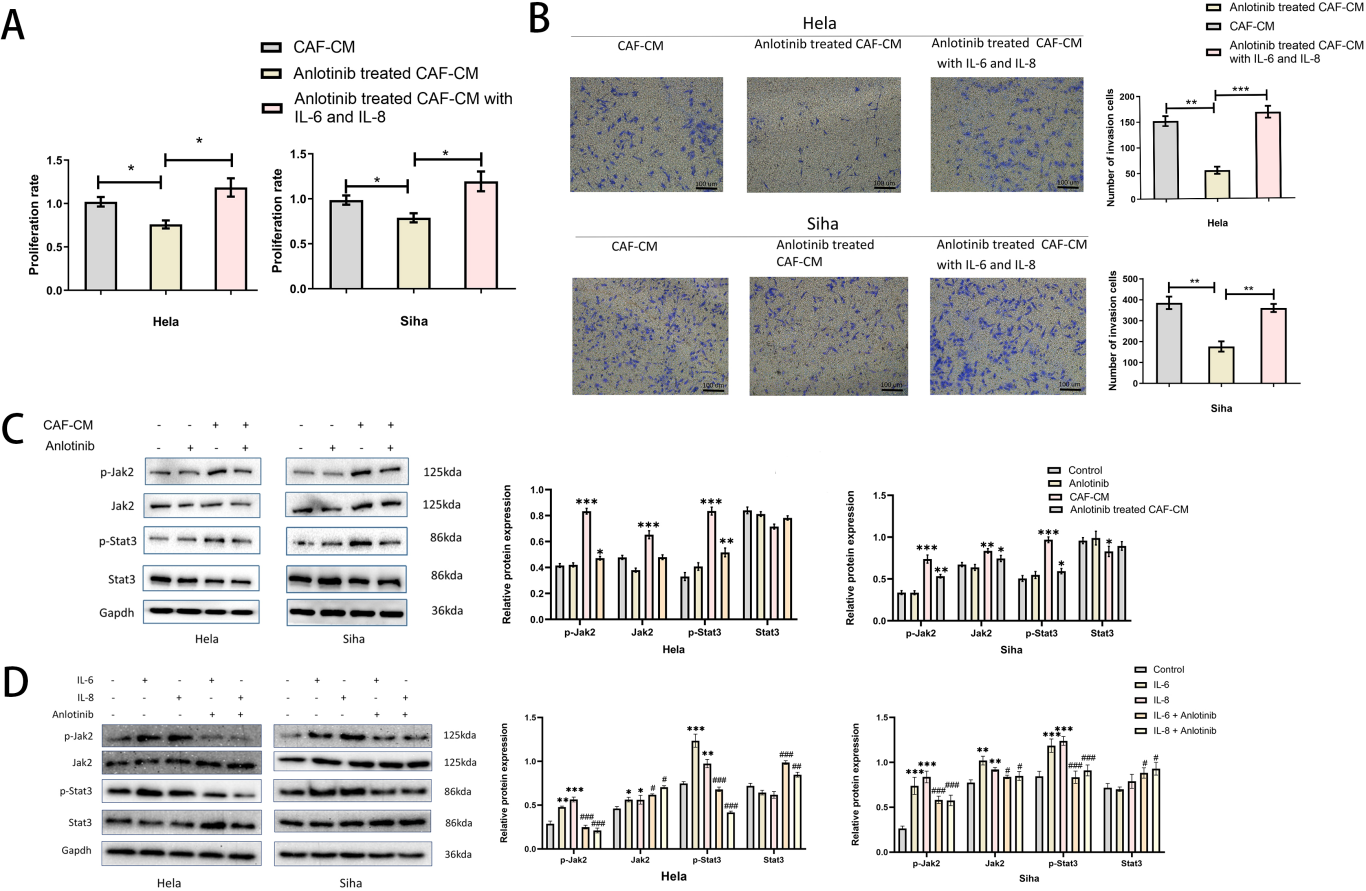
Anlotinib works by inhibiting the secretion of IL-6 and IL-8. **(A)** The effect of anlotinib-treated CAF-CM on cervical cancer cells proliferation was restored by the addition of recombinant IL-6 and IL-8 (n = 5). **(B)** The effect of anlotinib-treated CAF-CM on cervical cancer cells invasion was restored by the addition of recombinant IL-6 and IL-8 (n = 5) (Scale bar: 100um). **(C)** The effects of CAF-CM and anlotinib-treated CAF-CM on the phosphorylation of Jak-2 and Stat3 in Hela and Siha cells were evaluated by western blotting. CAF-CM increased the phosphorylation of Jak-2 and Stat3 in cervical cancer cell lines, but the treatment of anlotinib attenuated this promotion(n=3). **(D)** The effects of IL-6 and IL-8 on Jak-2 and Stat3 phosphorylation in Hela and Siha cells were evaluated by western blotting. IL-6 and IL-8 were able to promote the phosphorylation levels of Jak-2 and Stat3 in Hela and Siha cells, and anlotinib can neutralize this promoting effect(n=3). *P<0.05, **P<0.01, ***P<0.001. Compared with the control group. #P<0.05,##P<0.01,###P<0.001. Compared with the IL-6 and IL-8 group.

To clarify the specific effects of IL-6, IL-8 on cervical cancer cell lines, we added human recombinant IL-6 and IL-8 proteins to anlotinib-treated CAF-CM and found that it restored the promotional effects on the proliferation and invasion of cervical cancer cell lines (**Figures 7A, B**). In addition, we confirmed the effects of CAF-CM and anlotinib-treated CAF-CM on the phosphorylation of Jak-2 and Stat3 proteins, which are widely recognized as representative downstream targets of several cytokines, including IL-6 and IL-8. CAF-CM increased the phosphorylation levels of Jak-2 and Stat3 in cervical cancer cell lines, however, treatment with anlotinib attenuated this promotion (**Figure 7C**). In addition, we also demonstrated that recombinant IL-6 and IL-8 proteins can promote the phosphorylation of Jak-2 and Stat3 in cervical cancer cell lines, and anlotinib can neutralize this promoting effect. (**Figure 7D**).

### 3.7 Anlotinib can inhibit the proliferation of cervical cancer tumors in animals, and the combination treatment with docetaxel inhibits tumor proliferation more significantly

We investigated the effects of anlotinib on cervical cancer tumors in animals and in combination with docetaxel in animals (docetaxel is one of the standard therapeutic agents targeting cervical cancer in the clinic), and we carried out the treatment modalities of anlotinib and docetaxel, both alone and in combination, in a nude xenograft model of Siha+CAFs (**Figure 3A**). Although both treatment with anlotinib alone and docetaxel alone inhibited tumor proliferation, the combination of anlotinib and docetaxel inhibited tumor growth to a greater extent than monotherapy (**Figures 3B, C**). In terms of safety, there was no significant difference in body weight between the groups (**Figure 3D**). Immunohistochemistry results showed a significant reduction in the number of Ki-67-positive cells within the monotherapy groups of anlotinib and docetaxel as well as the combination of the two compared to the control group, but the reduction was more pronounced in the combination group (**Figures 3E, F**). Similarly, α-SMA-positive regions were significantly reduced in both the anlotinib and docetaxel monotherapy groups compared with the control group, and the inhibition of α-SMA expression was more pronounced in the combination group (**Figures 3E, F**).

### 3.8 Anlotinib can inhibit the expression of inflammatory factors in animal xenograft tumors and promote the apoptosis of tumor cells

**Figure 8:**
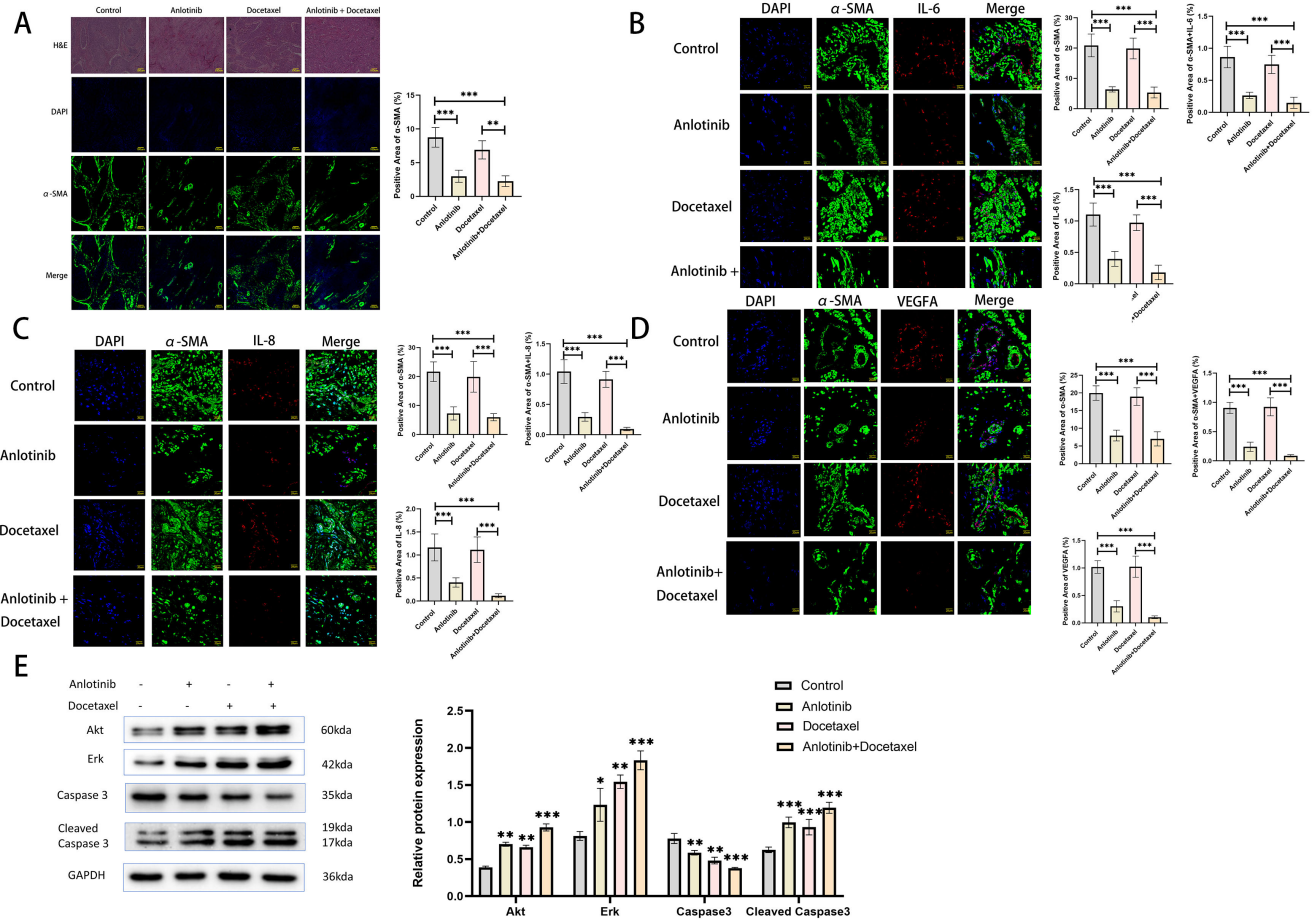
Anlotinib inhibits the expression of inflammatory factors in tumor tissues and promotes tumor cell apoptosis. **(A)** Representative photographs of H&E staining and immunofluorescence staining at low magnification for the four treatment groups(n=5)(Scarl bar: 100um). **(B)** Representative images of immunofluorescence staining was used to detect the expression of α-SMA and IL-6 in the four treatment groups(n=5)(Scarl bar: 20um). **(C)** Representative images of immunofluorescence staining was used to detect the expression of α-SMA and IL-8 in the four treatment groups (n=5)(Scarl bar: 20um). **(D)** Representative images of immunofluorescence staining was used to detect the expression of α-SMA and VEGFA in the four treatment groups(n=5) (Scarl bar: 20um). **(E)** Representative bands for western blot analysis of Akt, Erk, Caspase3, and Cleaved Caspase3 in tumor tissues from the four treatment groups(n=3). *P<0.05, **P<0.01, ***P<0.001. Compared with the control group.

In order to investigate the effect of anlotinib on inflammatory factors in xenograft tumors, we examined the expression of inflammatory factors in tumor tissues of the four treatment groups. The results showed that the expression of inflammatory factors was mainly concentrated in the α-SMA-positive regions, and the treatment of anlotinib could inhibit the expression of IL-6, IL-8, and VEGFA as well as the expression of α-SMA in these regions, whereas the treatment of docetaxel alone could not inhibit the expression of IL-6, IL-8, VEGFA, and α-SMA. Treatment with anlotinib in combination with docetaxel had a more pronounced inhibitory effect on IL-6, IL-8, VEGFA and α-SMA compared with the treatment group alone (**Figures 8B–D**). To investigate the effect of anlotinib on tumor cell apoptosis, we examined the expression of apoptosis-related proteins (Akt, Erk, Caspase3, Cleaved Caspase3) within the four treatment groups, and the results showed that compared with the control group, both anlotinib and docetaxel treatments alone led to an increase in apoptosis, with a more pronounced effect in the combination treatment group (**Figure 8E**).

## 4 Discussion

Through this study, we found that CAFs can promote the proliferation of cervical cancer cells both *in vivo* and *in vitro*, and that treatment with anlotinib inhibits the ability of CAFs to promote the proliferation of cervical cancer cells. This study is the first to investigate the role of anlotinib in inhibiting cervical cancer cells by inhibiting CAFs. Previous studies have shown that CAFs can secrete a variety of cytokines, including IL-1β, IL-6, IL-8, IL-10, IL-11, TGF-β, VEGFA, TNF-α, and VCAM-1, and through this study we have found that the expression of these cytokines can be inhibited by anlotinib.

In recent years, there has been a growing interest in the role of the TME in promoting tumor progression, with a large number of research efforts focusing not only on cancer cells, but also on other cell types of the TME, thus aiming to expand and identify new therapeutic options. Fibroblasts represent a heterogeneous family of cells composed of many subtypes whose activation can alter immune cell composition, promote or inhibit tumor growth, construct pre-metastatic niches, or stabilize blood vessels. These effects can be achieved through cell-to-cell interactions that form the extracellular matrix or through the secretion of cytokines or chemokines (15, 16). Activation of the PDGF/PDGFR and FGF/FGFR signaling pathways has been reported to significantly induce the proliferation and activation of fibroblasts (17). Anlotinib is a novel oral multi-targeted TKI that effectively inhibits VEGFR, PDGFR and FGFR. Previous studies have found that anlotinib can alleviate hepatic fibrosis in rats by down-regulating the expression of α-SMA and type I collagen in hepatocytes. It can also significantly improve lung function and inhibit lung tumor growth in a mouse model of lung adenocarcinoma combined with pulmonary fibrosis. In this study, we found that anlotinib inhibited the expression of activated CAFs and α-SMA, thereby reducing the invasiveness of cervical cancer cells. Based on these findings, targeting fibroblasts and inhibiting activated CAFs may become a strategy to suppress tumor progression (15).

In this study, a low concentration (4 uM) of anlotinib cannot inhibit the proliferation of NFs and cervical cancer cell lines (**Figures 4A, B**), but specifically inhibited CAFs activation, α-SMA and FAP expression (**Figures 4A, C, D**) and cytokines expression and secretion (**Figures 6B, C**) *in vitro*, thus suggesting that anlotinib is more potent against CAFs than cervical cancer cells more effectively.

In the present study, we identified that anlotinib inhibited the activation of CAFs and significantly suppressed the expression of several cytokines, especially IL-6, IL-8, VEGF and TNF-α. These cytokines have been reported to play important roles in tumor development. For example, IL-6 regulates Jak2/stat3 cell transduction, which promotes value-addition, invasion, and immunosuppression in a variety of tumors (18–21). IL-8 has been found to be involved in tumor cell proliferation, invasion and metastasis in gastric, lung and breast cancers (22–24). VEGF is an important factor that promotes angiogenesis and is involved in tumor proliferation and angiogenesis. TNF-α plays an important role in all stages of tumor development and is also involved in the functional regulation of the tumor microenvironment and immune resistance (25, 26). From these findings, it appears that anlotinib may be able to inhibit the promotion of cervical cancer cells by CAFs by inhibiting these cytokines.

In this study, we found that anlotinib inhibited the secretion of IL-6, IL-8 and VEGFA by CAFs. IL-6, IL-8 have been reported to induce chemoresistance in cancer through activation of the Jak2/Stat3 signaling pathway (27), activation of Stat3 can further promote VEGF expression and induce tumor angiogenesis (28, 29). In this study, we found that IL-6 and IL-8 could promote the phosphorylation levels of Jak-2 and Stat3 proteins in cervical cancer cells. And anlotinib not only inhibited the secretion of pro-cancer cytokines induced by CAFs, but also inhibited the phosphorylation of Jak2 and Stat3 in cervical cancer cells induced by CAFs. Addition of human recombinant IL-6 and IL-8 to CAF-CM after anlotinib treatment restored their promotional effects on proliferation and invasion of cervical cancer cells. This suggests that it is possible that anlotinib exerts its inhibitory effect on cancer cell development by inhibiting the Jak-2/Stat3 signaling axis.

Cancer is characterized by up-regulation of pro-angiogenic factors such as VEGFA, FGF, PDGF and angiopoietin and down-regulation of anti-angiogenic factors such as thrombospondin, vasopressor and endothelial inhibitor, leading to neovessel formation and tumor progression. Targeted angiogenic therapy combined with chemotherapy is currently a clinical solution for refractory cervical cancer (30). Clinical studies, represented by GOG240, have demonstrated that the use of bevacizumab, an agent that targets VEGFR, in combination with chemotherapy significantly improves progression free survival (PFS) and overall survival (OS) in patients with advanced cervical cancer (31). Previous studies have demonstrated that anlotinib has a favorable effect of inhibiting angiogenesis and reducing microvessel density (32, 33). A phase II clinical study demonstrated that anlotinib alone has favorable efficacy and safety profile in recurrent and metastatic cervical cancer (34). Several studies have found that anlotinib in combination with paclitaxel-based chemotherapeutic agents has shown good efficacy in advanced esophageal, pancreatic, and ovarian cancers (35–37). In this studies, both anlotinib and docetaxel administered alone inhibited xenograft tumor growth and the proportion of Ki-67-positive cervical cancer cells within the tumor. Docetaxel is a cytotoxic analog frequently used clinically for the treatment of recurrent and metastatic cervical cancer. And the combination of anlotinib with docetaxel can more significantly inhibit the tumor growth and the proportion of Ki-67-positive cells *in vivo* (**Figure 3E**). Docetaxel inhibits cellular micro-tubule protein polymerization and blocks the mitotic process, and anlotinib inhibits the pro-proliferative ability of CAFs and targets tumor angiogenesis. Combined, one directly kills cancer cells and the other targets the TME, which synergistically inhibits the growth of xenograft tumors. In addition, we examined the effect of anlotinib on α-SMA in *in vivo* experiments. The results showed that anlotinib significantly inhibited α-SMA expression *in vivo*, and the inhibitory effect could be enhanced in combination with docetaxel (**Figures 3E, F**, **8A**).

In order to investigate the effects of anlotinib on inflammatory factors within xenograft tumors, we examined the expression of IL-6, IL-8 and VEGFA in tumor tissues of four treatment groups by immunofluorescence staining. The results showed that the expression of these inflammatory factors was concentrated in the α-SMA-positive region, suggesting that they were mainly secreted by CAFs. Treatment with anlotinib inhibited the expression of inflammatory factors, whereas treatment with docetaxel was not able to inhibit the expression of inflammatory factors, but the combination of the two showed a stronger inhibitory effect (**Figures 8B–D**). To investigate the effect of anlotinib on tumor cell apoptosis, we examined representative proteins related to apoptosis such as Akt, Erk, Caspase3 and Cleaved Caspase3 in tumor tissues of the four treatment groups, and the results showed that both anlotinib and docetaxel treatments alone promoted tumor cell apoptosis, and the combination of the two showed a more significant effect (**Figure 8E**). Previous studies have also found that anlotinib can promote tumor cell apoptosis through Erk, BTK, PI3K/AKT/mTOR signaling pathways (38–40).

Of course, there are some limitations in this study. Firstly, anlotinib can inhibit several signaling targets including VEGFR, PDGFR and FGFR, and we could not confirm the role of these targets in this study. Secondly, only the expression and secretion levels of some common cytokines were examined in this study, and other cytokines were not examined, so we could not confirm the role of other cytokines in the development of cervical cancer cells. Thirdly, how anlotinib works synergistically with docetaxel in *in vivo* experiments needs to be explored in more studies. In the future, more studies are needed to explore and confirm.

## 5 Conclusions

In conclusion, this study demonstrated that anlotinib could inhibit the pro-cancer effects of CAFs by suppressing the activation of CAFs and the secretion of various pro-cancer factors. Anlotinib may be a useful tool for regulating activated CAFs and pro-cancer cytokines. In addition, we found that the combination of anlotinib and docetaxel acted synergistically and significantly reduced tumor volume in a xenograft tumor model. This suggests to us that the combination of anlotinib and docetaxel may be a potential strategy for the treatment of refractory cervical cancer.

## Data Availability

The original contributions presented in the study are included in the article/supplementary material. Further inquiries can be directed to the corresponding author/s.
